# Intergenerationality Programs—Between Children and Older Adults—For Portuguese Population: A Scoping Review

**DOI:** 10.3390/nursrep12040081

**Published:** 2022-11-14

**Authors:** Maria Inês Carvalho, Maria João Póvoa, Mariana Neves, Joana Bernardo, Ricardo Loureiro, Rafael A. Bernardes, Inês F. Almeida, Elaine Santana, Rosa Silva

**Affiliations:** 1 The Health Sciences Research Unit: Nursing (UICISA:E), Nursing School of Coimbra (ESEnfC), Avenida Bissaya Barreto, 3004-011 Coimbra, Portugal; 2 Portugal Centre for Evidence-Based Practice: A JBI Centre of Excellence, 3004-011 Coimbra, Portugal

**Keywords:** aged, healthy aging, intergenerational relations

## Abstract

The aging process is characterized by diverse and complex changes in the individual’s various dimensions, requiring continuous adaptation. In this sense, this transition can be faced from an active aging standpoint through strategies such as intergenerationality programs/projects, resulting in an active social participation and valorization that is so important to life in society. This review aimed to map existing programs/projects to promote interaction between children and older adults in Portugal to understand the extent and type of evidence available. A scoping review was developed guided by the JBI methodology and using PRISMA-ScR. The studies included six programs/projects promoting intergenerationality identified in Portugal, focusing their actions on promoting active aging and preventing problems associated with aging. The evaluated dimensions along the implementation of these programs were in the cognitive, motor, emotional and communicational domains, including parameters such as self-esteem, self-confidence, self-worth, well-being, loneliness and depression. These programs/projects present themselves as potential senior mental health promoters. However, other dimensions have been evaluated during these programs’/projects’ applications.

## 1. Introduction

Like other countries, Portugal has undergone demographic changes in recent decades, significantly transforming its age structure and populational dimension. In 2021, the aging index was 182.1% [1]. At the same time, according to the National Institute of Statistics (INE), there is a predicted decline in the number of young people, from 1.5 million to 0.9 million, and an increase in the number of seniors, from 2.1 to 2.8 million, doubling the aging index, from 147 to 317 older adults for every 100 young people in 2080 [2].

From a biopsychosocial perspective, the aging process is ruled by various complex transformations, losses and limitations on different levels compared to other life cycle phases [3,4].

In this sense, aging can be defined as natural, slow, progressive, continuous, multidimensional, multidirectional and dynamic, affecting all of our organism’s organs and tissue, leading to an inevitable increase in dependency, fragility and vulnerability, as well as the decline in seniors’ functionality [5,6].

On the other hand, aging is a role and behavior-changing process at a social level. In the same way, this process is ruled by multiple losses in this context, such as the loss of a valued social place as a productive individual and the loss of family and social and economic roles, driving the older adults to situational transitions, often leading to vulnerability, resulting in isolation, loneliness, depression, an increase in alcohol consumption, pathological anxiety and even suicide [7,8,9]. The absence of life projects and the lack of acknowledgement of the end of family social and parental responsibilities can effectively generate feelings of fragility, incapacity, uselessness, low self-esteem, dependency, loneliness, hopelessness and existential emptiness [10,11].

It appears that factors such as social exclusion, stigma, unemployment, poverty and economic situation affect mental health and increase the need to intervene among this population, which is increasingly needed and suitable [12,13].

This way, since aging is a period marked by losses and gains, the individual must adapt to the developmental transition process, accompanied mainly by other situational and health-illness transitions [4,7,11]. Consistent well-being will result from balance when experiencing the transition, allowing the individuals to develop their physical and psychological capabilities [14,15].

In this sense, according to literature based on some studies developed on the subject, intergenerationality programs/projects seem to be one of the active aging promotion strategies, encouraging mental health promotion, capable of initiating continuous and positive changes during the various phases in people’s life cycle [6,8,10].

Thus, as aging is a period marked by losses and gains, the individual must adapt to the developmental transition process, accompanied mainly by other situational transitions and health-disease [4,7,11]. Consistent well-being will result from the balance in experiencing transition, allowing individuals to develop their physical and psychological capabilities [14,15].

Therefore, successful aging will be one in which the person can experience life transitions and reach well-being. Within a multidisciplinary team, the challenge for nurses, given the need for support in transition processes, is to understand the transition process itself and implement interventions that provide effective help to people to provide stability and a sense of well-being [10]. Nursing care for older adults must be based on a multidisciplinary and multidimensional perspective, understanding the aging process of people, maximizing its potential, reducing dependencies and increasing the quality of life, and nursing interventions that promote interactional activities that enhance well-being and social inclusion, fighting isolation and sedentary lifestyle are crucial [10,11].

According to the literature based on some studies developed on the subject, intergenerational programs/projects are thus considered effective interventions for inclusion and cooperation. These inclusive methodologies, based on these two phenomena, become relevant as they prioritize the social value of equality and seem to be seen as one of the strategies to promote active aging, encouraging the promotion of mental health, capable of triggering continuous and positive changes during the various stages of people’s life cycles [6,8,10].

According to the World Health Organization (WHO) [16] and the Directorate-General for Health (DGH) [17], active aging is understood as a process of optimizing health, participation and safety to improve the quality of life as people age, maintaining functional capacity and well-being.

This type of relationship is expected to be increasingly common in the following years because one of the goals of the 10th Sustainable Development objective for 2030 advocates empowerment and promotion of social inclusion [18]. This way, cooperation between generations could be a potential strategy, encouraging contact between people of different generations to be increasingly frequent.

Intergenerationality is a social interaction between people of different ages and generations through exchanging life experiences, values and principles [6,13,19]. Nowadays, expanding these relationships for the whole of society becomes essential as an incentive for intergenerational solidarity [6,20].

The growth of older adults brings social changes between generations, which are evident in the loss of spaces to give and receive affection, share and communicate due to the accelerated dynamics of modern societies, focused on the production, development and consumption of goods and services [10,20].

So, intergenerational practices can be defined as a way to unite people with a purpose, resorting to activities that mutually benefit them and promote better respect and understanding between generations [6,21]. Faced with the reality of the aging world population, international organizations have increased and promoted being oriented towards formulating policies that contribute to coexistence between generations [19]. This implies expanding the concept of intergenerationality, considering it not only as coexistence between groups of individuals of different ages but valuing both the scope and the importance of each generation itself and the contribution that the interrelation between them offers to individuals, the community and the society [10,19].

This symbiosis between generations allows the achievement of joint goals and articulates interests. Then defined, the relationships between generations must be characterized by the presence of a series of elements that favor the production of «intergenerational synergy». In short, whenever in the relationship between generations, actions and behaviors are capable of repercussions on our environment and, in turn, bringing benefits to it, we can speak production of intergenerational synergy [11,20,21].

In this sense, it was considered pertinent to carry out a Scoping Review (ScR) since it allows us to find available evidence regarding this specific area of investigation, also clarifying the conceptual limits of the topic under study [22,23].

The present ScR’s objective is to find programs/projects existent in Portugal, promoting interaction between children and older adults, and identify the evaluated dimensions, including those in the mental health field, among the older adult population, during its implementation.

In this sense, the following review question was formed: “What programs/projects promoting generational interaction between children and older adults exist in Portugal?”.

To complement the research and analysis of the available evidence, the following specific review questions were formed: (1) “What are the characteristics of these programs/projects?”; (2) “Which dimensions in the older adults population were evaluated during these projects’/programs’ implementation?”; (3) “Which of these dimensions relate to the mental health of the older adults population?”; and (4) “What is the perception of professionals and older adults about intergenerational programs/projects?”.

## 2. Materials and Methods

The present ScR was based on the methodology recommended by JBI [23].

The PRISMA 2020 was also used [24].

### 2.1. Research Strategy

In an initial phase, to analyze and verify the language and most used terms in the literature related to this theme, as well as which descriptors are appropriate, a limited search of the database Academic Search Complete and CINAHL Complete via EBSCOhost was conducted using the keywords: older, child, intergenerational and Portugal in conjunction with the Boolean operator AND.

In the second phase, after the map of concepts to use was defined, we proceeded to the individualized search in the databases Academic Search Complete, CINAHL via EBSCOhost, MEDLINE via PubMed, MedicLatina, via EBSCOhost and RCAAP (the RCAAP portal is an aggregator (meta-repository) that collects the description (metadata) of documents deposited in various institutional repositories, research data repositories and scientific journals in Portugal).

The search strategy was adjusted and adapted to each database. That way, for CINAHL Complete, we used CINAHL Subject Headings, and MEDLINE Completed, we used the Medical Subject Headings (MeSH) descriptors. In the third and last phases, the bibliographic references of the selected studies were analyzed. The complete search strategy used in CINAHL Complete is schematized in Table 1.

The inclusion criteria for this ScR were based on the PCC mnemonic: Population, Concept and Context, being that (i) the population corresponds to the people older than 60 years old (institutionalized or living in the community) and children younger than 10 years old (it should be noted that based on the definition, according to the DGH (2022), that adolescence is the period between 10 and 19 years old, and to exclude adolescents from the study population, the age group of children under the age of 10 years old was defined as a component of the population under analysis); (ii) the concept is programs/projects promoting intergenerationality; iii) all potential intergenerationality practice configurations and geographical contexts in Portugal. Studies in English, Portuguese and Spanish were included.

We defined exclusion criteria as: (i) published studies that only identify intervention results for children and adolescents; (ii) studies containing carried out programs/projects that do not include at least one healthcare or social professional; and iii) studies focusing their research on parenting interactions.

### 2.2. Data Extraction

All attained articles were exported to the Mendeley V1.19.8 (Mendeley Ltd., Elsevier, The Netherlands) software, where they were recovered, stored and duplicates were eliminated. After this operation, we read the title and abstract, applying our exclusion criteria. The complete article text was thoroughly evaluated with our inclusion criteria by two independent reviewers, with the information congruent with our objective and review questions being oriented by an instrument developed by the authors and carried out independently [23,24]. The extracted data will be presented in a more detailed form, with tables and figures and descriptive narration of the results, as recommended by ScR’s directives [23].

## 3. Results

**Figure 1:**
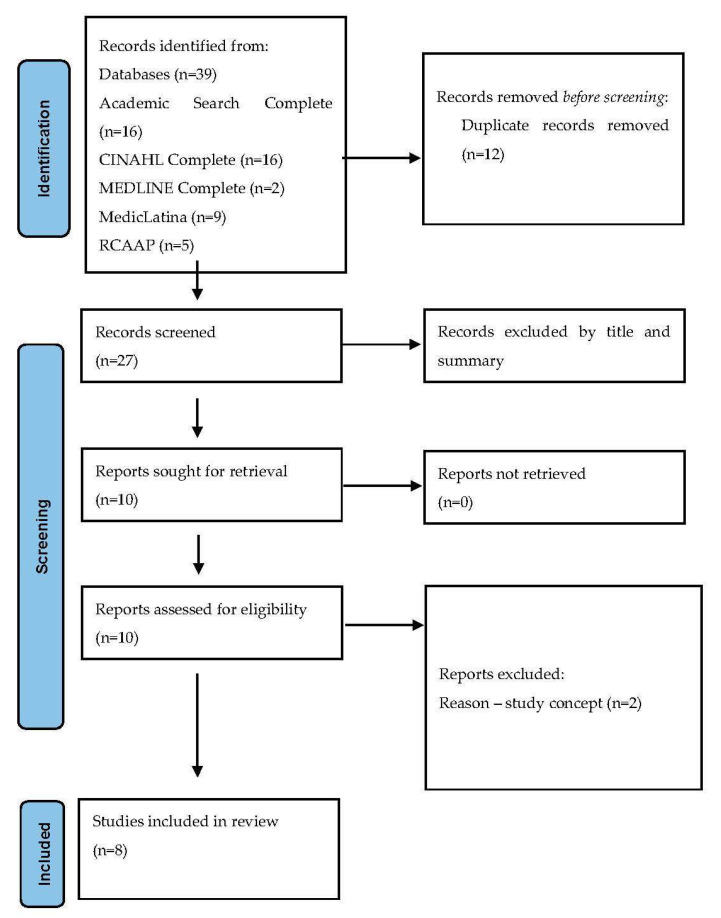
PRISMA flow diagram of the systematic review process [24].

Through research in the databases mentioned above, and after applying the inclusion and exclusion criteria, we obtained seven articles in the Academic Search Complete database, sixteen articles in CINAHL Complete, two articles in MEDLINE Complete, none in MedicLatina and five studies in RCAAP (grey literature resource), making up a total of thirty-nine studies (Figure 1).

Eight studies were selected, four in Portuguese [25,26,27,28] and four others in English [29,30,31,32]. Out of these included studies, one fits in the quantitative paradigm [32], three in the quantitative paradigm [26,27,29], three have a mixed approach [25,30,31] and one is a systematic review of the ScR type [28].

This section intends to analyze the selected studies, emphasizing their results. In this sense, we will synthesize these programs’ characteristics in the scope of intergenerationality, as presented in the analyzed studies (Table 2 and Table 3).

### 3.1. Applied Program/Project Characteristics

By analyzing the included programs/projects, 50% of these are integrated into previously existing programs/projects that seek to promote other areas beyond intergenerationality—combined programs [26,28,29], making up the remaining 50% we have programs/projects focused on intergenerationality alone—we called them projects without combined intervention, meaning they are only focused on this area of intervention [25,31,32]. Generally, these programs’/projects’ intervention periods varied between two and twelve months of fieldwork, having between two and twenty-six total sessions, each lasting from 15 to 120 min [25,26,28,29,31,32], see Table 2. As for the population inserted in those programs/projects, the age of the older adult population ranged from 64 to 97 years old, with the ages of children varying from 3 to 13 years old [25,26,28,29,31,32]. It is worth noting that in some studies, the age group of the study population is not specified [26,28,32]. For other studies, the upper age limit established for the juvenile population was surpassed to allow for their inclusion, as these studies were relevant for understanding the matter. So, the maximum age was considered 11 years old in the studies of Valente (2015) and Barbosa (2020) [25,31] and 13 years old in the study of Henriques (2021) [32].

In order to answer our review questions, namely the characteristics and perception of professionals and older adults in these programs/projects, we decided to include two studies (presented in Table 3), as we consider that they aggregate relevant and pertinent to the results about the meaning of the older adults who participate in these programs [30] and the professionals who implement attributes to the theme [27].

### 3.2. Evaluated Dimensions in the Applied Programs/Projects

The evaluated dimensions in the multiple programs/projects were organized into five categories: emotional, motor, cognitive, communicational and interactional. In each category, there were multiple evaluated aspects (Table 4). These categories were performed a posteriori, i.e., after floating readings of the results extracted from the articles. Regarding the evaluation of the activities carried out, only three studies applied satisfaction surveys at the end of their sessions, showing “interesting” or “very interesting” results [25,26,28]. Other studies applied surveys and scales to assess dimensions such as self-esteem, loneliness and self-worth [29,31,32] (see Table 2).

## 4. Discussion

The nursing intervention should function as a process that facilitates the experiences of transitions experienced by older adults, promoting maximum autonomy and well-being.

**Programs/projects promote generational interaction between children and the older adults**

Six of the eight studies refer to programs/projects implemented within the scope of intergenerationality [25,26,28,29,31,32]. The remaining studies [27,30] address the perceptions of older adults and professionals regarding intergenerational relationships/practices.

Thus, in response to the central question of this ScR, we verify that, in Portugal, there are at least six programs/projects implemented within the scope of intergenerationality, which were subjected to investigation and consequent dissemination of the results.

**The characteristics of these programs/projects**

Overall, the programs/projects analyzed have a heterogeneous structure of the design, implementation and evaluation (assessed dimensions and evaluation moments), with theoretical and practical sessions held for both generations. In these sessions, the target population’s interest and motivation were parameters considered by all studies, using expository, interactive and interrogative methods.

With this mapping and analysis, we can verify that several themes were addressed, demonstrating that these intergenerational practices enable a multivariate and comprehensive approach focusing on active and healthy aging.

The sessions’ duration is heterogeneous, lasting from 15 to 120 min. Thus, the recommended amount of time the older adults and children should share to obtain good results is between 45 and 90 min, according to Soares [27]. Some of the analyzed studies do not present a duration within this range [26,29,31]; we can consider that there is not an overall consensus between professionals regarding the ideal duration of these activities. This duration will certainly also depend on the type of activity being developed. This is because cognitive activities will require more concentration/attention from these groups. So, the duration should be well thought out, while other activities, such as more recreative ones, can be longer for older adults and children.

**The evaluated dimensions in the older adult population during the implementation of these programs/projects**

The theory and available evidence seem to demonstrate that Intergenerationally can be seen as one of the strategies that promote active aging, capable of triggering positive and continuous changes during the various phases of people’s life cycles [19,20].

Older generations primarily transmit knowledge to younger generations, essential for preserving collective culture [25,29,31].

The dimensions evaluated in the various programs/projects were organized into five categories: emotional, motor, cognitive, communication and interaction.

Within each category, several parameters were evaluated. In the emotional aspect, parameters such as satisfaction, affection, well-being, depression and self-esteem were assessed. The evaluated aspects show positive results, as shown in Valente [25], which showcases the presence of affection, happiness and satisfaction in the participants in all the sessions, who considered that the activities developed foster dialogue and Intergenerational coexistence. The themes related to the emotional and interaction elements are transversal to all selected programs/projects [25,26,28,29,32].

Vieira et al. [29] show positive results regarding well-being and happiness, as the older adults describe their time spent on activities with children as joyful and something that makes them feel well. In this study, it is also possible to see benefits in terms of intergenerational learning since children would teach seniors how to play with a tablet. In turn, seniors taught children to play board games. Besides this, in terms of communication, their mutual understanding was notorious because, in group activities, both the children and the older adults enjoy finding ways to understand each other using verbal and non-verbal communication.

Estevam [26] also highlights positive results in terms of positive emotions, adding to these the presence of social benefits, given that for older adults, working or supporting school garden activities reveals that besides therapeutic benefits, these activities also contribute to social inclusion. So, the results of Barbosa et al. [31] have also shown that the intervention caused a significant reduction in loneliness and depression, being also relevant in terms of self-esteem and a sense of rejuvenation, since for older adults, the opportunity to share life experiences enhances these feelings, providing the construction of an affective bond. Another point highlighted simultaneously in this study and Henriques [32] demonstrates that the older adults’ active participation in planning activities to be carried out allows them to feel a sense of purpose in life with the establishment of goals.

The study by Pascoal et al. [28], in line with the results of the other studies included in this review, also points out that it is possible to see positive results in terms of increasing literacy on what active aging and intergenerational activities consist of, and that, on the other hand, interacting with children is a facilitating factor in their life processes/transitions, for example, when acquiring coping strategies in the grieving process. Another factor evidenced as a potentiator of the older adults’ adhesion to intergenerational activities was the possibility of them being able to transmit knowledge and values to the children with whom they related in developing the activities of the programs/projects.

In the same way, younger generations can also transmit knowledge and promoters of older adults’ well-being, social participation and self-valorization [26,28,29,31,32].

In addition to the studies included here, other studies also support this idea [6,21,33]. In this sense, intergenerationality is perceived by Estevam [26] and Henriques [33] as a principle that promotes the mental health and the quality of life of older adults, as well as equality between generations, enabling the transformation of mentalities. On the other hand, it also contributes to fostering citizenship, which, in turn, should facilitate inclusion, social solidarity and people’s well-being. This idea is supported by Cantinho [34]. In the same line of thinking, this same strategy admits the promotion of mental health as one of the areas of intervention for active aging, which is as important as the promotion of physical health in the older adult population, if not more. In this context, the scientific evidence analyzed alerts us to the importance of environments that favor well-being and mental health and enable older adults’ autonomy and independence [11,15].

So, the development of activities that promote intergenerationality and allow older adults to be an integral part of it seems to have therapeutic effects that will contribute to the maintenance of the person’s functionality and, consequently, to healthy aging [26,28]. In this way, several authors indicate that programs focused on promoting solidarity between generations present benefits for younger and older adults, obtaining gains from this experience [13,20]. Even so, comparing the results from intergenerational relationships perceived by institutionalized older adults [25,26,29,31,32] and older adults people residing in the community [28,30], it was possible to verify differences. Generally, both groups point to positive points such as satisfaction, affective bonds and feeling of reward. However, the older adults residing in the community still need to set boundaries, stating that, despite finding it exhausting to take care of children, they feel a moral obligation to take care of their grandchildren as a matter of family support.

This reality highlights the impact they can have in different areas, particularly in the mental health area, and the gains that can result from this at a motor, cultural and emotional level, reinforcing the added value of their implementation. In the daily clinical practice of nurses.

According to the results of all the studies analyzed, these gains are positively reflected in older adults at a cognitive level, changes in mood and vitality, as well as self-concept, in their autonomy, their social image of old age, as well as having a preventive action within the scope of isolation, through the social inclusion of the older adults, promotion of physical and mental well-being and respect for the younger generations, fostering both linguistic and digital learning [25,26,27,28,29,30,31,32] In children, the gains will be reflected in their social behavior, avoiding antisocial behavior and the valuation of the aging process, as well as a greater acceptance of im-age alteration and demystification of old age, thus reducing stereotypes and derogatory comments [25,26,27,28,29,30,31,32]. These practices influence their values and cultural learning, thus promoting a better adaptation to the contexts of personal and professional life in their future [27,32], an idea shared by other studies [6,21,34,35].

Thus, including older adults in this panorama of joint and cooperative activities allows them to obtain benefits related to psychological factors of elementary importance, such as self-esteem and emotional well-being. It also allows them to promote their quality of life, receive social benefits and oppose social paradigms associated with their capabilities [26]. As an example, the implementation of the horticultural program, as can be seen in the results mentioned above, proved to be a significant social factor, as it integrates older adults and contradicts a paradigm of disability associated with them, thus acting as a promoter of social inclusion [26]. This way, one begins to simultaneously minimize loneliness and isolation, which are very much associated with the older adult population, serving as a stimulus to the possibility of having active aging [4,11].

**Limitations and implications for scientific research**

This review’s limitation is that only studies were considered in Portuguese, Spanish and English.

On the other hand, given that scoping reviews do not seek to assess the methodological quality of the studies included for analysis, recommendations for clinical practice cannot be issued [36]. Despite this, some limitations of the included studies were reported to provide valuable information to future research studies.

Bearing in mind the potential benefits mentioned above and being aware of their importance, we focused our research on the existence of programs/projects carried out in Portugal, something that in itself constitutes a limitation of our study, as these projects/programs were not also studied in an international dimension, which would undoubtedly bring another type of designs and interventions.

We note that there is already scientific evidence, albeit still very incipient, on the contribution and impact of implementing projects and programs within the scope of intergenerationality, despite the methodological weaknesses of the studies developed.

Therefore, this is still an area of little investment and evolution, with limited empirical results. This limitation may result from the fact that there are programs/projects in the community in this area which are not associated with a scientific research component, making it impossible to locate them in the different databases, thus compromising the rigorous and effective mapping of the available evidence. This way, and given that intergenerational practices represent a topic with current relevance in research, the elaboration of new studies can contribute essentially to implementing new programs/projects. We consider that it is essential to invest in this area and that these studies may show the potential benefits of these activities for the older adult population as well as what the role of nurses in this type of activity is, since, in the total number of employees specified in each study, there was no mention of any nursing professionals. We believe that if the effectiveness of this intervention is not studied, the replication of this practice in context will undoubtedly be conditioned. Therefore, we need rigorous and credible evidence from the development of experimental studies and economic studies that explore the respective cost–benefit and contribute to supporting the practical realization of this type of intervention.

## 5. Conclusions

Aging is a life cycle phase that requires continuous adaptation to the environment and the emergence of biological, psychological and social changes. In this way, technological developments and changes in family structures in today’s society have influenced the lives of older adults. On the one hand, they have allowed an increase in the average life expectancy but, on the other hand, have not ensured the necessary support for it to be experienced with quality, causing harmful consequences in the motor, cognitive and emotional dimensions.

However, this life transition can be seen from the perspective of active aging and intergenerationality through the implementation of policies and strategies that encourage and stimulate the interaction of the older adults with younger people so that both generations acquire knowledge of their potential and their limits, thus producing benefits, through the transmission of learning and experiences, resulting in an appreciation and active social participation that are significant in life in society.

Given the above, the relevance of this topic that we decided to address became evident. The realization of this ScR made it possible to critically analyze the results of the selected studies according to the previously established criteria, verifying their congruence, robustness and consistency. Through the analysis and discussion of the results, we were able to obtain relevant conclusions to answer our research question and objectives that were initially outlined, as well as to suggest the urgency of strengthening research in this specific area, with repercussions for the clinical practice of the nursing discipline, policymakers and health researchers.

## Figures and Tables

**Table 1:** The complete research strategy used in CINAHL Complete.

Search	Query	Records Retrieved
**#1**	TI (aged or “aged people” or “aged 65+” or elder* or geriatric* or old * or “old people” or “older adults” or senior*) OR AB (aged or “aged people” or “aged 65+” or elder* or geriatric* or old* or “old people” or “older adults” or senior*)	13,991
**#2**	TI (“kids” or “child*”) OR AB (“kids” or “child*”)	10,874
**#3**	TI (“intergenerational activities” or “intergenerational programs” or “intergenerational projects” or “intergenerational relations”) OR AB (“intergenerational activities” or “intergenerational programs” or “intergenerational projects” or “intergenerational relations”)	1
**#4**	TX (portugal or “portugal population” or “portuguese people”) OR AB (portugal or “portugal population” or “portuguese people”)	3115
**#5**	(MH “Aged”) OR (MH “Aged, 80 and Over”)	919,958
**#6**	(MH “Child”)	511,577
**#7**	(MH “Intergenerational Relations”)	6167
**#8**	(MH “Portugal”)	6531
**#9**	#1 OR #5	1,265,123
**#10**	#2 OR #6	782,285
**#11**	#3 OR #7	6219
**#12**	#4 OR #8	36,091
**#13**	#9 AND #10	154,870
**#14**	#11 AND #13	1117
**#15**	#12 AND #14	16

Note. A similar strategy will be used for the remaining databases.

**Table 2:** Synthesis of the program’s/project’s characteristics in the scope of intergenerationality.

Author, Year	Type of Program		Sessions and Interventions			Population
	Programs/Projects Without Combined Intervention	Programs/Projects with Combined Intervention	Number of Sessions	Session Duration	Intervention Period	Age Group/Average Age $\bar{\left(x\right)}$
**Valente (2015)** [25]	√		10	60 min	7 months	Children: [8 to 11 years old] Seniors: [65 to 95 years old]
**Vieira et al. (2016)** [29]		√	8	60 to 120 min	4 meses (2 conception and 2 intervention)	Children: [3 to 6 years old] Seniors: [74 to 96 years old]
**Estevam (2018)** [26]		√	26	<15 min	12 months	Children: [3 to 6 years old] Seniors: [not specified]
**Barbosa et al. (2020)** [31]	√		12	120 min	12 months	Children: [6 to 11 years old] Seniors: [72 to 90 years]
**Pascoal et al. (2020)** [28]		√	2	Not specified	2 months	Children: [non specified pre-school age] Seniors: [64 to 84 years old]
**Henriques (2021)** [32]	√ (Including other programs in this one)		10 activities. It is not specified how many were carried out in each session)	Not specified	12 months	Children: [9 to 13 years old] Seniors: [65 to 97 years old] Inmates: [Not specified]

**Table 3:** Perception of professionals and older adults about intergenerational practices and relationships.

Author, Year	Study Design	Sample	Concept	Context	Results
**Humboldt, S., Monteiro, A., & Leal, I. (2018)** [30]	Qualitative study, in-depth interviews were conducted.	316 Seniors of three different nationalities (Portuguese, German and English), with an average age of 71.2 years old.	The study was based on the analysis of interviews meant to identify the older adult’s perspectives on intergenerational relationships with their grandchildren.	Community Centers in Lisbon metropolitan area and Algarve region.	The attained results were grouped into six subthemes: *(1)* Affection and reward; *(2)* Interest and integration; *(3)* Interaction quality between grandfather and grandson.; *(4)* definition of privacy and limits; *(5)* support provision; *(6)* the obligation to support grandchildren.
**Soares, G. (2018)** [27]	Qualitative study, in which the data collection was carried out using narrative and semi-structured interviews.	29 professionals, of which 90% were women and 10% were men (psychologists, assistants, social professionals, animators sociocultural (with/without higher education), gerontologists and educators), performing functions in social, educational and political organizations.	The “Intergenerational Practices” theme was analyzed from professionals intervening in the social area and/or intergenerational professionals through 29 interviews and direct observation registered in field notes.	Municipal and social organizations and schools.	Obtained data were divided into four axes, *(1)* “Experiences in the institution and multidisciplinary work”; *(2)* “Level of involvement, influence and limits—self and the other”; *(3)* “Evaluate: essential or expendable?” *(4)* “The feeling of return, scepticism and hope”.

**Table 4:** Categories of evaluated dimensions in the applied programs/projects.

Study		Emotional	Motor	Cognitive	Communicational	Interactional	Evaluation Type	Evaluation Moment
	Evaluated Dimension							
**Valente (2015)** [25]		- Satisfaction; - Affection; - Happiness.	-	-	- Intergenerational Dialogue.	- Conviviality between generations; - Sharing of experiences and values.	Satisfaction surveys	After each session
**Vieira et al. (2016)** [29]		- Well-being; - Happiness.	-	- Intergenerational Learning.	- Mutual communication and understanding.	- Interaction between generations.	Interviews with ten children and five older adults; surveys with ten parents	After completing the program implementation
**Humboldt et al. (2018)** [30]		- Affection and feeling of reward; - Ambivalence of intergenerational relationships; - Provision and obligation to support grandchildren; Feelings of uncertainty.	-	-	-	- Interest and integration; - Quality of grandparent-grandchild interaction; - Privacy and limits.	Interviews with older adults	Not applicable
**Estevam (2018)** [26]		- Positive emotions (happiness); - Satisfaction.	-	-	- Positive words and expressions and gratitude.	- Complicity; - Social benefits (social inclusion).	Satisfaction surveys	During and after each session and after complete program implementation
**Barbosa et al. (2020)** [31]		- Loneliness; - Depression; - Self-esteem; - Happiness; - Joy; - Well-being; - Satisfaction; - Feeling of rejuvenation; - Construction of affective bond; Setting goals.	-	- Setting goals.	-	- Sharing of experiences with the institutional community.	Subjective Happiness Scale; Rosenberg Self-esteem Scale; Geriatric Depression Scale; UCLA Loneliness Scale; Child Depression Inventory; Loneliness Scale	Before and after the complete program
**Pascoal et al. (2020)** [28]		- Gratitude; - Well-being; - Satisfaction; - Way of facing life; - Grief processes.	-	- Changes in acquired knowledge.	-	- Sharing of experiences and values.	Satisfaction surveys	After completing the program implementation
**Henriques (2021)** [32]		- Self-confidence; - Self-appreciation.	-	- Self-awareness.	- Communication between generations.	- Inclusion in the community; - Receptiveness to change.	Surveys	Before and after completing the program implementation

## Data Availability

Data sharing is not applicable to this article.
